# Perceived Pain in People Living with Amyotrophic Lateral Sclerosis—A Scoping Review

**DOI:** 10.3390/nursrep14040220

**Published:** 2024-10-17

**Authors:** Debora Rosa, Laura Ingrande, Ilaria Marcomini, Andrea Poliani, Giulia Villa, Martina Sodano, Duilio Fiorenzo Manara

**Affiliations:** 1 Center for Nursing Research and Innovation, Faculty of Medicine and Surgery, Vita-Salute San Raffaele University, 20132 Milan, Italy; rosa.debora@unisr.it (D.R.); l.ingrande@studenti.unisr.it (L.I.); marcomini.ilaria@unisr.it (I.M.); poliani.andrea@unisr.it (A.P.); manara.duilio@hsr.it (D.F.M.); 2 IRCCS Istituto Auxologico Italiano, 20149 Milan, Italy; m.sodano@auxologico.it

**Keywords:** chronic pain management, daily activities, nursing, quality of life, social relationships

## Abstract

(1) Background: Pain is a common symptom in patients with Amyotrophic Lateral Sclerosis (ALS). There are no evidence-based pharmacological treatments for pain in ALS; recommendations are based on guidelines for chronic non-oncological pain and clinical experience. The aim is to map the literature on how people with ALS experience pain, and how this affects their daily activities and social relationships. (2) Methods: This scoping review included studies concerning patients with spinal/bulbar ALS aged ≥ 18 years who experience pain, focusing on perception, characteristics, treatment, and impact on quality of life. Temporal and linguistic criteria were applied when searching the MEDLINE, CINAHL, and SCOPUS databases. (3) Results: The management of pain in these patients is complex and involves the use of anti-inflammatory drugs, analgesics, and opioids. Pain is associated with other conditions such as depression and anxiety, which contribute to a deterioration in the quality of life. Moreover, pain may also negatively influence patient compliance with prescribed treatment regimens and the quality of care they perceive themselves to be receiving. (4) Conclusions: It is of the most importance to identify effective ways to assess and treat this issue, with health care professionals taking an active role in this process.

## 1. Introduction

Amyotrophic Lateral Sclerosis (ALS) is a progressive disease caused by motor neuron degeneration in the spinal cord and brain, resulting in paralysis and usually death by respiratory failure [1]. The onset of ALS usually manifests as limb weakness (spinal onset) or difficulty speaking or swallowing (bulbar onset). In particular, the onset of ALS is more common among men in the 70–80 age group. Annually, ALS affects approximately 2 to 3 people per 100,000 [2]. The median age at ALS diagnosis is 65.2 years (IQR 56.0–72.2 years) for men and 67.0 years (IQR 59.0–74.0 years) for women [3]. Between 58 and 82% of people with ALS have a spinal onset [4]. The most recently available data, for the average incidence rate in the United States, is reported as being 2–3 per 100,000, with an age-adjusted prevalence of 6.6 per 100,000. There has been an increase in this rate in recent decades [5]. In Europe, the incidence of ALS is between 2.1 and 3.8 per 100,000 people [3].

ALS is a multi-system disease characterised by the presence of early and diverse non-motor symptoms. Non-motor symptoms, such as cognitive impairments [6], behavioural disturbances [7], autonomic dysfunctions [8], and sensory/painful disorders [9], play a significant role in the disease’s clinical presentation. These manifestations not only contribute to the overall burden of the disease, but may also serve as important indicators of its progression and impact on patient survival.

Until the past decade, pain in ALS was largely overlooked by clinicians. Nevertheless, the substantial impact on the quality of life of ALS patients and their caregivers necessitates the identification and evaluation of this issue. Recent ALS treatment guidelines have indicated that pain may be a presenting symptom in ALS patients and should, therefore, be treated [10]. However, given the paucity of empirical evidence on this topic, pain in ALS is frequently underdiagnosed and inadequately addressed [2,10]. Although the mechanism underlying pain in ALS remains poorly understood, a substantial body of research indicates that pain prevalence among patients with this condition exhibits considerable variability, with estimates ranging from 15% to 85% [11]. Such conditions are commonly associated with and a consequence of various factors, including muscle contractures, reduced joint mobility, muscle cramps, spasticity, and pressure on the skin due to prolonged periods of immobility [12,13]. Moreover, although the World Health Organization (WHO) definition of palliative care places a strong emphasis on the importance of pain relief measures, there is currently no evidence-based pharmacological treatment for pain in ALS. Indeed, the recommendations for treatment are based on guidelines for chronic non-oncological pain, which are then combined with the clinical experience of treating patients with ALS and pain [14]. This scoping review aims to provide an overview of research investigating how patients perceive pain and its impact on activities of daily living, as well as social relationships and adherence to the treatment pathway.

## 2. Materials and Methods

The objective of a scoping review is to map and summarise disparate forms of research evidence (quantitative and qualitative), which illustrates the breadth and depth of a given concept or phenomenon within its disciplinary or professional context [15].

This scoping review has been conducted in accordance with the JBI Manual for Evidence Synthesis [16] and in accordance with the PRISMA ScR checklist [17] for its construction. Prior to the writing of the review, a planned search was conducted on PROSPERO and the JBI Review Register to ensure that there are no ongoing review protocols on this topic.

The primary research question guiding this review was as follows: What is the impact of pain in ALS patients?

Secondary questions were identified that may be useful in outlining how the evidence was mapped. These included the following.

1. How do ALS patients perceive pain?
2. What are the characteristics of pain perceived by ALS patients?

The research question was structured according to the Participant-Problem/Concept/Context (PCC) Framework, as proposed in the ‘JBI Manual for Evidence Synthesis,’ in order to guide and direct the development of specific eligibility criteria, keywords, and search strategies [16] (Table 1).

### 2.1. Eligibility Criteria

#### 2.1.1. Inclusion Criteria

The selection of studies for this review was based on studies that responded to the formulated research question and the PCC Framework (Table 1) [16]. Therefore, studies that focused on patients diagnosed with spinal or bulbar ALS and aged ≥18 years were selected. Furthermore, studies discussing the experience of the perception of unpleasant sensations (i.e., pain, discomfort), characteristics, frequency, treatment, and its impact on patients’ quality of life were included.

No limitations were imposed regarding geographical location, and the language filter was set to English and Italian.

#### 2.1.2. Exclusion Criteria

Trials were excluded if the following characteristics existed. Document Types: commentaries, letters, and editorials. Study Subject: Studies conducted on patients with diseases other than ALS, except where such patients were used as control groups. Research Focus: documents investigating the perceptions of caregivers or family members. Publication Types: grey literature, due to the lack of peer review.

#### 2.1.3. Filters

The databases were subjected to certain pre-processing filters, which included the imposition of language filters for English and Italian, as well as a temporal filter limiting the search to documents published within the last decade.

### 2.2. Research Strategy

A three-step search was conducted in accordance with the JBI guidelines [16]. The initial stage of the search entailed a preliminary exploration of at least two databases: The databases MEDLINE via PubMed and Cumulative Index to Nursing and Allied Health Literature (CINAHL) were searched. A second search was conducted utilising keywords and free terms on all identified databases: MEDLINE via PubMed, CINAHL, and SCOPUS. Finally, a search was conducted on the bibliographies of the full texts of the articles identified in the previous two steps. The keywords employed were “pain,” “sensory perception,” and “amyotrophic lateral sclerosis,” with the Boolean operators AND and OR utilized. All identified literature sources were exported into Mendeley, a bibliographic source management software. The search strings for the 3 databases yielded 138 articles. In the final step, 13 articles were selected for further analysis. These articles were read, and their results are reported in detail in a data extraction table. One author (L.I.) conducted the data extraction, with two other authors (D.R. and M.S.) subsequently verifying the results independently. The electronic database search was conducted from July 2022 until May 2024.

### 2.3. Article Selection

#### Data Extraction

**Figure 1:**
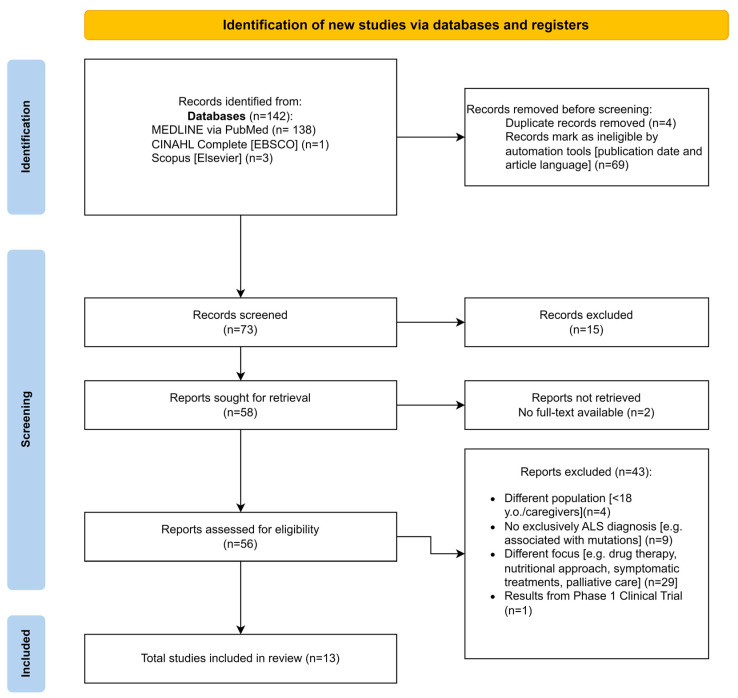
PRISMA flow diagram.

The PRISMA Flow Diagram 20 [18] was followed throughout the selection process. In the first phase, titles and abstracts were screened by two reviewers (L.I. and M.S.) and assessed according to the inclusion criteria (Figure 1).

In the second phase, the full text of the selected articles was evaluated in detail by the same reviewers, always using the same inclusion criteria.

The reference manager Mendeley v. 2.100.0 was used to systematically collect, sort, and de-duplicate all records and data. Metadata were extracted using the Mendeley reference manager and manually checked on import. Data were extracted from the selected documents using a data extraction table. This table consisted of the following elements: author/year, purpose, population, study design, type and duration of intervention, pain assessment tools (including interviews), and outcomes.

## 3. Results

### 3.1. Study Characteristics

Figure 1 shows the selection process that led to the selection of thirteen articles. Two of these are secondary literature articles: a narrative review [4] and a systematic review and meta-analysis [19]. Furthermore, one exploratory qualitative study was included [20] (Table 2 and Table 3).

Of the thirteen articles included, ten are observational studies, including six with a cross-sectional design [13,14,21,23,24,25], one RCT [11], one cohort study [22], one case–control study [26], and one prospective observational study [2]. The selected studies were published between 2012 [11,22] and 2022 [4]. Three are Italian [11,22,23], and three are English [19,24,26]. The remaining studies are two Swedish [14,20] one German [13], one Japanese [21], one Chinese [2], one Korean [4], and one exploratory qualitative study [20]. In terms of sample size, one study [20] had a sample size of fewer than 20 patients, six studies [13,14,21,22,23,26] included 40 to 80 patients, one study included 636 patients [24], and three studies [2,11,25] included 160 to 197 patients.

### 3.2. Data Collection Tools

The most frequently used instruments for data collection in the selected papers are (Table 4): the Brief Pain Inventory (BPI) [28], used in six studies [2,11,13,20,25,26], the ALS Functional Rating Scale (ALS-FRS-R) [29], used in five studies [2,13,14,23,25], and the face-to-face interview [2,11,20,24] and the Hospital Anxiety and Depression Scale (HADS) [30], used in two studies [24,25].

### 3.3. The Way Pain Is Expressed

In five [4,13,20,22,26] of the thirteen articles in the review, this aspect is the focus of the authors’ attention.

Nagging, sore, periodic, annoying, enduring, debilitating, worrying [22], and exhausting [13,22] were the adjectives most frequently used by patients. Other adjectives used were crampy [13], intense, soft [26], unbearable, crushing [13], electric [26], searing, dull [13], piercing, throbbed, cold, screamed [19], stinging [4,20], and sharp [4,26]. No subject described their pain as pounding or tearing. Only in one study did a few patients use the terms crushing and oppressive as descriptors [22]. In some interviews [20], patients described their pain experience with the phrases ‘very intense, like cramp, very bad,’ ‘like stiffness, heavy feeling,’ ‘It’s like sharp needles of pain all the time,’ and ‘pain that weakens.’

### 3.4. Pain Intensity

The Brief Pain Inventory (BPI) is the most used validated measurement scale [28]. Findings from the utilisation of this scale indicate that more than half of the population analysed report moderate to severe pain [11,13,14,25,26]. The same results are found in the systematic review and meta-analysis [19], in which a total of 1426 participants were included. Of these, 78.8% classified their pain as moderate, while 17.5% classified it as severe [19].

In alignment with these findings, the mean score obtained using the Pain Detect Questionnaire scale [40] within the Wallace et al. [26] study yielded an average pain rating of 3.7. Conversely, the results of the Pizzimenti et al. [23] study exhibited a considerably higher average pain score. In this case, the visual analogue scale (VAS) [34] was employed, and the results revealed an average pain intensity of 7.6 (standard deviation ±2.7). 11 of the 36 patients indicated that their pain was the most intense imaginable. Additionally, 14 patients described their pain as moderate, severe, or very severe. A single individual classified their pain as “mild.”

We then identify the NRS numeric scale [24]. In this paper, 625/636 (98.3%) completed the scale, with 429 (68.6%) reporting pain, with an average score of 2 [24]. Additionally, the Italian Pain Questionnaire (QUID) [38] utilized within the Italian study conducted by Pagnini et al. [22] is worthy of mention. Of the 40 participants, 19 reported experiencing pain. Of these, 10 (47.6%) described it as ‘moderate’ [22].

The study by Åkerblom et al. [14] utilises the Pain Severity Index, in conjunction with the mean of the three items from the Brief Pain Inventory (BPI) [28], with a view to determining the intensity of pain experienced by patients. The results of this analysis indicate that 8 of the 16 patients categorise their pain as ‘moderate.’

In a subsequent study [2], discordant results are observed in relation to the Pain Severity Index. In this study [2], participants were divided into two groups: an ALS patient group and a control group comprising peripheral neuropathy patients. In the first group (N = 89), the majority of patients (24) described their pain as “mild,” 9 as “moderate,” and 2 as “severe.” In the control group, comprising 89 participants, 8 patients described their pain as “mild,” 10 as “moderate,” and 2 as “severe” [2].

In a study by Ishida et al. [21], the Wong–Baker Faces Pain Rating Scale (WBS) [36] was employed. The authors [21] selected this scale for use in the study because the majority of participants exhibited significant communication difficulties. In this study, the correlation was found to exist between intensity and the functional status of the patient. The reported outcomes demonstrated that subjects with full dependence exhibited significantly higher levels of pain intensity [21].

### 3.5. Pain Localisation

A review of the literature revealed that the most reported locations of pain were the upper and lower extremities [4]. In particular, the most frequent sites were the shoulders and upper extremities, followed by the lower extremities and their associated joints and muscles [11,13,21,23].

The incidence of head/neck/trunk/back pain was relatively low [4,19]. In contrast, Kong et al. [2] and Wallace et al. [26] present results that are in discordance with those presented in the aforementioned studies. In Wallace et al. [26], pain was reported in the following areas with the highest prevalence: legs, arms, shoulders, neck, back, feet, abdomen, and hands. In contrast, Kong et al. [2] reported that pain was most frequently reported in the head and abdomen (neck, back, shoulders, and chest). These authors report that in their study sample, pain primarily occurred in the distal limbs. Furthermore, eight patients reported pain in multiple areas. Åkerblom et al. [14] highlight that pain may manifest in various areas of the body, including the eyes. It is not uncommon for patients to experience pain in a particular body region, but this can also extend to multiple areas, with potential for gradual spread to different regions [14].

### 3.6. Pain Management

From the analysis of the thirteen articles, only six [3,11,12,20,25] of them address the topic of pain management in ALS patients. Wallace et al. [26] indicate that of the 42 participants, 54% utilised regular analgesia, while 29% engaged in regular opioid usage. However, the specific drugs used are not specified [26]. The most commonly used drugs are non-steroidal anti-inflammatory drugs (NSAIDs) [11,13,14], analgesics [14], opioids [13,14,21], spasmolytics, and triptans [14]. One patient reported taking Ketadolone [13], while two patients (3%) did not receive any medication for pain management [14]. In the Italian study by Chiò et al. [11], non-steroidal anti-inflammatory drugs (NSAIDs) were the most commonly used drugs for the treatment of pain, with or without the concomitant use of opioids.

Furthermore, muscle relaxants, antidepressants, and other drugs are employed, including pregabalin [4,11,21], quinine sulphate [4,11], and gabapentin [4,11]. First-line treatment typically involves non-opioid analgesics, while opioids are employed when these drugs are ineffective in relieving pain [21]. For the treatment of muscle cramp pain, quinine sulphate and magnesium are frequently employed; for spasms, baclofen, diazepam, and lorazepam; and, finally, for musculoskeletal pain, non-steroidal anti-inflammatory drugs (NSAIDs), paracetamol, tramadol, and morphine [4]. As reported by Stephens et al. [25], the majority of clinicians involved in managing pain are neurologists, followed by general practitioners. Approximately 30% of the study participants did not receive any pain treatments. On average, 2.9% of patients in the study received palliative care, which was not specified [25].

### 3.7. The Impact of Pain on Activities of Daily Living (ADLs)

Regarding the impact of pain on activities of daily living (ADLs), the analysis of studies reveals a lack of consensus. Although Stephenson et al. [25] found that the intensity of pain did not influence the extent to which it disrupts an individual’s daily activities, Wallace et al. [26] demonstrated that the level of pain interference in daily living activities is correlated with pain intensity. Despite demonstrating that pain intensity does not correlate with its interference in ADLs, one study [25] indicates that it is not the severity of the pain itself that determines a patient’s activity level. In addition, pain is considered the main cause of the deterioration in quality of life in ALS [11,26], although this may be due to comorbid depression [23]. One study [14] examined participants’ satisfaction with various aspects of their daily lives, rather than how pain interferes. The same researchers conducted an exploratory study one year prior [20], in which they interviewed a sample of 16 patients. Most participants in the study reported a loss of autonomy due to pain. This necessitated assistance with everyday tasks such as clothing self-care and domestic chores [20].

### 3.8. The Impact of Pain on Social Relationships

The findings indicated that pain during social activities resulted in limitations in the daily lives and leisure time of the participants, who described themselves as inactive. Some participants ceased engaging in activities that provoked pain: “So now I sometimes feel that I don’t want to do certain things because I know that then I will get a stab of pain” [20]. In certain instances, the patients’ friendships and social lives were negatively influenced by pain [20]. This was exemplified by one participant who described how pain became an obstacle to being able to continue walking with friends. This participant had trouble walking due to pain in his leg, which made it challenging for him to keep up with the group. Eventually, this became impossible [20].

### 3.9. The Relationship between Pain and Other Pathologies

The studies reviewed indicate that there is a significant relationship between pain and other pathologies, and that emotions are highly integrated with the experience of pain, particularly negative emotions, which often worsen the perception of pain [20].

Pain may also contribute to the experience of low mood and emotions such as fear, anxiety, and the feeling of not being able to control or understand pain. Additionally, it can lead to a loss of self-esteem. Participants reported that pain caused psychological distress when it reinforced a sense of imperfection and a feeling of helplessness. This occurred, for example, when they found themselves unable to handle a task as expected or to avoid difficult situations due to the anticipation of stronger pain [20].

Further research indicates that pain is associated with a decline in quality of life and a greater prevalence of depression [21]. Depression and anxiety are common in this type of patient, and pain, anxiety, and depression affect several domains of quality of life. It has been proposed that anxiety frequently co-occurs with depression, which may result in an overestimation of the impact of anxiety on quality of life, when in fact, depression has not been considered [24,25].

The study conducted by Pizzimenti et al. [23] yielded contrasting results. It was found that the duration and frequency of pain were not significantly related to depression or quality of life (QoL) scores. Furthermore, pain intensity did not significantly influence depression scores. Conversely, both pain intensity and depression scores demonstrated significant correlations with diminished quality of life [23].

### 3.10. Interference with Compliance and Standard of Care

One study [4] mentions pain related to conditions that are not directly linked to the pathology, but are a consequence of it. The aforementioned examples include pain resulting from prolonged immobility, which can lead to pressure on the skin and the potential development of pressure ulcers. Another source of pain is face masks, particularly those applied to the nasal bridge, when used on patients undergoing long-term, non-invasive ventilation (NIV). Additionally, tube stasis may also lead to discomfort, particularly in patients undergoing invasive ventilation via tracheostomy.

Nevertheless, none of the studies cited make any mention of the issue of poor compliance among patients. The authors emphasise the necessity of reaching a consensus on the timing, intervals, and content of pain assessments in ALS. Furthermore, the authors propose the creation of specific guidelines for the management and treatment of pain in ALS. They argue that the successful treatment of pain can improve the quality of life of patients and their caregivers, even in the absence of a cure for the disease [4].

### 3.11. Pain and Palliative Care

It can be observed in the vast majority of articles [20,21,26,29] that consider the pain experience of patients diagnosed with Amyotrophic Lateral Sclerosis (ALS), a common element is found: the insufficient attention paid by professionals around these patients to the identification, detection, and management of pain. Pain is a significant symptom present throughout the trajectory of the disease and is associated with significant morbidity [26]. Furthermore, it is a symptom that is present throughout this disease [19]. Ishida et al. [21] emphasise that pain is regarded by patients as the most distressing symptom in the final stages of the disease. Additionally, patients with reduced functional status often encounter communication difficulties, which may result in inadequate pain reporting or a complete lack of reporting. Furthermore, some participants appeared to be resigned to their pain, indicating a lack of resilience and motivation to seek help from health care professionals [20].

## 4. Discussion

This scoping review aims to provide an overview of the ways in which individuals with ALS perceive pain and the extent to which it impacts activities of daily living, social relationships, and adherence to the care pathway, despite the definition of palliative care.

### 4.1. Pain Perception

Regarding pain perception, each patient experiences and describes or names pain differently [42]. Furthermore, the fact that multiple descriptors for pain have been selected by patients implies that the individual experiences a range of pain symptoms that represent a combination of underlying pathological processes [26]. Moreover, pain in ALS is not only nociceptive or mechanical; it can also have neuropathic and sensory dimensions [11]. This multidimensional aspect encompasses sensory disturbances that have been recently demonstrated through skin biopsy studies, revealing the degeneration of small nerve fibres in ALS patients [9]. These sensory changes, which are often overlooked, suggest that ALS not just is a motor neuron disease, but also involves a sensory component. It can be concluded that pain experiences are primarily recurring and periodic in the lives of these patients. Indeed, many patients have become resigned to their pain and do not even report it to their health care providers [14,20,43].

This underscores the necessity for physicians and health care professionals to be educated on the significance of pain and other non-motor symptoms of this disease [26], for assessments to be standardised [2], for pain to be promptly identified, for a multidisciplinary approach to be employed in the management of this disease, and for therapy to be administered in a timely manner to relieve pain, particularly in patients with end-stage ALS [2] and with decreased functional status [21,44].

Although standardised approaches to pain detection and quantification are valuable, flexibility in recording the specific attributes and experiences of pain is crucial, given the considerable inter-individual variability in pain presentation and perception [45]. This will assist in comprehending the disparate impacts of pain and in furnishing palliative care requirements that are tailored to the individual patient [19].

In their study, Åkerblom and colleagues [20] propose the tracking and quantification of pain episodes, including their duration, intensity, and frequency, in addition to pain-free periods and their duration. This monitoring would necessitate the development of modern assessment methods, which could be accomplished through the use of digital solutions and the application of artificial intelligence [20]. Such developments would facilitate the implementation of patient-specific, rather than generic, pain assessment methods, a crucial consideration given the diversity of pain experiences and their multifaceted nature [20].

### 4.2. Pain Intensity

Regarding the measurement of pain intensity, the selected literature reveals a notable heterogeneity in both the methodologies employed and the outcomes obtained. In summary, the perceived pain experience is described as moderate to severe [46]. Only one study was identified in which the results differed significantly, with participants either reporting pain as mild or even indicating the absence of pain [2]. Given the considerable divergences observed in the literature, it is challenging to compare the data and generalise the results.

### 4.3. Interference of Pain in ADLs and Social Relationships

The variability and fluctuation in the perceived intensity of pain is also linked to a variability in the interference it has on the activities of daily living and social relationships of these individuals [22,47]. Furthermore, the results are difficult to generalise due to conflicting evidence. While Stephens et al. [25] did not find a correlation between pain and interference in ADLs, other studies [11,13,26] have highlighted that pain in ALS is a significant factor in the deterioration of quality of life. Further studies [20,23] have demonstrated that this phenomenon may be attributed to the co-occurrence of depression [23]. With regards to social interactions, pain can impair patients’ social relationships and social activities. For instance, pain can hinder patients from maintaining their usual walking patterns with friends [20].

### 4.4. Pain Management

To cope with pain-related issues, patients who suffer from moderate to severe pain are commonly administered non-steroidal anti-inflammatory drugs (NSAIDs) [11,46]. These drugs represent the most common form of treatment for pain, often used in conjunction with opioids [11].

### 4.5. Localisation of Pain

The definition of pain localisation is similarly variable in its application [48]. It can, thus, be concluded that, in general, the majority of patients report experiencing pain predominantly in the upper extremities, including the shoulders and extremities [19], although Åkerblom et al. [14] highlight that pain may also affect various other areas of the body, including the eyes. It is not uncommon for patients to experience pain not in a single area, but rather, in multiple locations. Furthermore, the pain can gradually spread to different regions.

### 4.6. Areas for Improvement and Recommendation

In this review, we examine the perception of pain among individuals with Amyotrophic Lateral Sclerosis (ALS) and its impact on activities of daily living, social relationships, and adherence to the recommended treatment pathway.

A crucial area requiring enhancement is the attention devoted by health care professionals to the early detection and timely management of pain in these patients. To address this issue, some authors have proposed the implementation of standardised detection methods, potentially utilising digital solutions, to standardise the approach of all professionals in this regard.

The evidence suggests that patients with ALS may have problems with interoception, which is the body’s ability to sense and process internal signals like heart rate and breathing [49]. This can affect how they perceive and respond to pain, causing them to either downplay their pain or experience it differently. As a result, it may be harder to assess and treat their pain accurately. Interoceptive dysfunction could also impact their emotional reactions to pain, as the brain uses these internal signals when processing discomfort and other physical sensations [50]. Therefore, it would be helpful to conduct studies in order to deeply explore how changes in interoception may influence pain perception in patients with ALS [49]. Understanding these factors could lead health care professionals to use more sophisticated and comprehensive methods for evaluating pain. In this regard, new evaluation tools could be useful to better understand the level and the characteristics of pain among patients with ALS.

Additionally, investigating non-pharmacological interventions, such as physical therapy and cognitive behavioural therapy, may provide new approaches to alleviating pain by addressing sensory disturbances and interoceptive challenges in ALS patients.

No study tested a robust correlation between pain and ALS progression and its potential variations according to different ALS subtypes. Thus, further research should examine how pain presents differently based on ALS phenotypes and stages. This could help in developing more personalized and effective pain management strategies. Such a system would have a significant impact on assisting health care professionals in the management of pain by enabling the adaptation of therapy to the individual patient’s specific needs.

### 4.7. Limitations

It must be noted that this review is subject to several limitations. The extensive range of instruments employed for data collection renders a comparative analysis of the results obtained from the various studies a challenging endeavour.

A further limitation concerns the low quality of the papers included in the scoping review due to the cross-sectional nature of the studies. Longitudinal designs would make it possible to observe changes in pain over time, establish causal relationships, and reduce individual bias.

Articles written in languages other than English and Italian were excluded. This limitation may have excluded important information published in studies conducted in other languages, which may have limited the comprehensiveness of the review. These limitations and the characteristics of some studies may result in an inability to generalise the results.

## 5. Conclusions

A significant degree of heterogeneity emerges regarding the perceived sensation of pain by patients with spinal or bulbar ALS. Pain is a common occurrence in this disease, manifesting in various forms and exhibiting unpredictable patterns over the course of the disease [20]. However, it has a profound impact on the quality of life of those affected [13]. Given that the pain experienced by patients with Amyotrophic Lateral Sclerosis (ALS) is subject to variation in terms of its characteristics, including intensity, frequency, and location, there is a clear requirement for the development and implementation of methods of pain assessment and management within this patient population [20]. These methods can assist health care personnel in the recognition and effective treatment of this symptom, thereby improving the quality of life for these patients. It is of the utmost importance that all health care professionals who treat these patients are fully aware of the necessity of pain assessment and management, particularly given the evidence that many of them fail to report pain to the health care professionals responsible for their care. Therefore, it is essential that the initiative to address this issue comes from the care team itself [20].

## Figures and Tables

**Table 1:** PCC framework [16].

PCC	
**Population**	Patients (≥18 years old) with a diagnosis of spinal/bulbar ALS.
**Concept**	Pain
**Context**	No geographical limitations were imposed, ensuring that all contexts where people with ALS live and frequent are included, such as hospitals, communities, hospices, and homes.

**Table 2:** Data extraction.

Authors/Year	Aim	Sample	Study Design	Interventions	Results
Åkerblom et al., 2020 [20]	Exploring personal experiences of pain in people with primary lateral sclerosis	16 participants diagnosed with MND e.g., primary lateral sclerosis, with upper and lower motor neuron signs and symptoms	Exploratory qualitative study	In-depth interviews	Of the 16 participants recruited, 1 refused due to lack of interest in the study. The important findings were the experiences of unpredictability of pain outbreaks, the efforts required to manage pain, the consequences for activity and quality of life, and the suffering induced by decreased attention to and neglect of pain on the part of both patients and staff.
Åkerblom et al., 2021 [14]	To study associations between pain, disease severity, and individual quality of life (IQOL) in patients with primary lateral sclerosis	61 patients recruited from four multidisciplinary teams in Sweden, of whom 55 responded to the pain measure (The Brief Pain Inventory—Short form) and were included in the main analyses	Cross-sectional study	Disease severity was measured with the Amyotrophic Lateral Sclerosis Functional Rating Scale (ALSFRS-R), and individual quality of life was measured with study-specific SEIQoL-DW. Pain was measured with the Short Form of Brief Pain Inventory (BPI-SF).	Forty-one (74%) of the participants who responded to BPI-SF reported pain. Thirty-nine (71%) of those reported pain in the last 24 h. Pain severity was moderate on average, with eight participants (14%) reporting severe pain. Satisfaction with IQOL for the whole sample was good, and there was no difference in satisfaction with IQOL between those who reported pain/no pain. There was no correlation between severity of pain and satisfaction with IQOL, nor between severity of illness and satisfaction with IQOL.
Chiò et al., 2012 [11]	To assess the prevalence and characteristics of pain in an epidemiological series of patients with Amyotrophic Lateral Sclerosis (unspecified whether of spinal or bulbar origin) compared to population-based controls	160 ALS patients residing in the province of Turin; the controls were randomly selected from the lists of general practitioners.	Controlled population study	Interview and assessment of pain using the Brief Pain Inventory	Patients with ALS reported pain more frequently than controls, healthy subjects, with the same gender and age (±3 years). In ALS patients, pain was more frequently localised to the extremities.
Hanisch et al., 2015 [13]	To determine the prevalence, severity, interference, location, and type of pain experienced by 46 German patients with Amyotrophic Lateral Sclerosis (ALS) and to correlate this information with disease duration and severity parameters; to this end, the Brief Pain Questionnaire (BPI) was administered to the patients.	46 ALS patients and 23 controls with myotonic dystrophy type 2 (DM2)	Cross-sectional	Administration of the BPI and the ALS-FRS-R scale	78% of the 46 patients (n = 36) reported pain, compared to 54% of the controls. Patients with ALS, compared to controls, reported moderate to severe pain (42% vs. 20%). Pain in ALS patients interfered significantly more with daily activities than in controls.
Hurwitz et al., 2021 [19]	To determine the aggregate prevalence of pain in ALS, with respect to its measurement method and pain characteristics	Patients with Amyotrophic Lateral Sclerosis (not specified whether of spinal or bulbar origin)	Systematic review and meta-analysis	A critical examination of the pain measures employed; the measures were classified into three categories: validated (e.g., scales, questionnaires, and structured instruments), customised (e.g., interviews, single-question surveys, and multiple-question surveys), and customised with validated measures.	The overall prevalence of pain in all included studies was 60%, indicating that between half and two-thirds of all ALS patients experience pain. The most frequently reported site of pain in ALS patients is the upper extremities, although it may also manifest in other regions, including the lower extremities, head, back, and neck. The type of pain was frequently associated with cramps or spasms.
Ishida et al., 2018 [21]	Determining the frequency and characteristics of pain and its treatment in patients with ALS	80 patients diagnosed with ALS according to the El Escorial criteria were recruited for this study between 1 May and 31 May 2015.	Multicentre cross-sectional study	Administration of the Wong–Baker Faces Pain Rating Scale (WBS) and assessment of functional status using the Barthel Index	Pain was reported by 53.8% of ALS patients and 36.3% reported receiving painkillers. Opioids are the most commonly used drugs to treat pain. Differences in pain frequency according to functional status were not statistically significant (*p* = 0.38). Pain intensity in patients whose functional status was total dependence (BI 0–20, 2.5–1.2) was significantly worse than that in those with a better functional status (BI 21–60, 1.4–0.7; BI 61–99, 1.4–0.5; *p* < 0.01).
Kong et al., 2021 [2]	Studying the characteristics of pain in patients with ALS using standardised pain questionnaires	89 patients with ALS (not specified whether of spinal or bulbar origin) and 89 control subjects with peripheral neuropathy	Prospective observational study	In-person interviews with 89 ALS patients between January 2010 and June 2018; data including gender, age, ALSFRS-R scale score, and pain severity index (PSI) were collected. Characteristics were compared between ALS and peripheral neuropathy, and between ALS patients with and without pain.	There were no significant differences in the sex and age ratio between the two groups. There were many more patients with pain symptoms in the ALS group (35/89.39%) than in the peripheral neuropathy group (20/89.22%). Quality of life was significantly affected in ALS patients with pain (using ALS patients without pain as control subjects).
Kwak, 2022 [4]	To provide an overview of the epidemiology, clinical characteristics, underlying mechanisms, and management approaches to pain in ALS, with the objective of optimising clinical practice and patient outcomes		Narrative review		
Pagnini et al., 2012 [22]	Investigating pain in ALS patients and its influence on their quality of life (QoL)	40 patients with sporadic ALS, recruited from the Neuromuscular Omnicentre (NEMO) in Milan	Observational Study	Administration of the Italian Pain Questionnaire (QUID) and the McGill Quality of Life Questionnaire (MQoL) at time 0 and at follow-up after 4 months	Approximately half of the patients with ALS reported pain, which was described as bothersome, tiresome, and exhausting. These symptoms occurred intermittently, but persisted over time. Pain was found to be correlated with quality of life (QoL), and its intensity was found to be able to predict a worsening of QoL.
Pizzimenti et al., 2013 [23]	To assess the prevalence of pain in ALS patients, to compare depression and QoL measures in patients with and without pain, and to study the influence of depression and pain scores on the QoL of ALS patients with pain	Forty patients with ALS were enrolled, and thirty-six were included in the analysis.	Cross-sectional study	Quality of life (QoL) was assessed using the Quality of Life Index (QL- Index), depression using the Zung Self-Rating Depression Scale (SDS), and pain intensity using a visual analogue scale (VAS).	Twenty-six patients (72.2%) reported pain, localised as follows: scapulohumeral area (15 patients, 57.7%), lower limb (8 patients, 30.8%), and cervical–dorsal area (3 patients, 11.5%). The average pain intensity was 7.6 (±2.7), while the average duration of pain in the last 24 h was assessed as 1.4, which corresponds to approximately 2 h per day. The frequency of pain episodes in the last 24 h was 0.6 (approximately 2 per day). A total SDS score of 50 or higher, indicating depression, was found in only one patient. Sixteen patients self-reported several depressive symptoms, but remained in the non-depressed range (score between 35 and 47). Ten of the patients with a score of 35 or higher on the SDS were taking antidepressants.
Edge et al., 2020 [24]	To examine the prevalence of pain, anxiety, and depression in a large sample of people with ALS/ MND and to examine their interrelationships and effect on QoL, using a measure that recognises the multifaceted nature of QoL	636 participants with ALS/MND, diagnosed according to the criteria of the El Escorial World Federation of Neurology using a convenience sampling strategy	Cross-sectional study	Focus groups and 1:1 interviews; administration of the NRS numeric scale for pain assessment, the World Health Organisation Quality of Life Questionnaire (WHOQOL-BREF), and the Hospital Anxiety and Depression Scale for MND (mHADS)	636 persons with ALS, 69%, reported pain, 7% of the participants exceeded the published cutoffs for probable depression, and 14% had probable anxiety. Pain, depression, and anxiety all affect quality of life; depression has a significant effect on the physical and psychological domains of QoL, while pain affects physical QoL and psychological anxiety QoL.
Stephens et al., 2016 [25]	Exploring the relationship between depression, anxiety, self-efficacy, and the experience of pain in ALS patients	The patients included individuals registered with the Agency for Toxic Substances and Disease Registry (ATSDR) who indicated that they wished to be informed about the research studies.	Cross-sectional study	Online, anonymous public survey; ALS Functional Rating Scale-Review (ALSFRS-R); Brief Pain Inventory-Short Form (BPI); Hospital Anxiety and Depression Scale (HADS); and Chronic Pain Self-Efficacy Scale (CPSS)	197 participants responded to the survey. Borderline cases of depression and anxiety were common. Average pain levels were moderate. Higher pain self-efficacy scores predicted less pain severity, less pain interference and greater pain relief with treatment. As depression scores increased, pain interference with daily life was greater. In conclusion, anxiety and depression are common in patients with ALS and pain. Self-efficacy seems to attenuate pain.
Wallace et al., 2014 [26]	To gather information regarding pain in ALS using standardised questionnaires	42 patients with ALS; the control subjects included 41 healthy volunteers and 42 patients with neurological problems other than ALS.	Observational case-control study	Pain data were collected using The Brief Pain Inventory and The PainDetect Questionnaire.	85% of subjects with ALS reported pain compared to 50% of controls with neurological problems other than ALS and 35% of healthy controls (p < 0.01). Pain in ALS included cramps, aches, and fatigue, and was non-neuropathic. Pain had a significant impact on mood, general activity, relationships, and overall enjoyment of life. Of those with painful ALS, 54% used analgesic drugs regularly and 29% used opiates on a regular basis. Other non-motor symptoms included fatigue, constipation, urinary problems, itching, and drowsiness.

**Table 3:** Study characteristics.

Authors	Country	Sample	M/F	Severity of Disease ALSFRS-R † or Stage
Åkerblom et al., 2020 [20]	Sweden	16	11/5	32.50
Åkerblom et al., 2021 [14]	Sweden	61	39/22	37
Chiò et al., 2012 [11]	Italy	160	91/69	28.70
Edge et al., 2020 [24]	United Kingdom	636	390/246	_
Hanisch et al., 2015 [13]	Germany	46	20/26	33.10
Hurwitz et al., 2021 [19]	United Kingdom	_	_	_
Ishida et al., 2018 [21]	Japan	80	45/35	_
Kong et al., 2021 [2]	China	178	54/35 ALS 57/32 peripheral neuropathy (control group)	_
Kwak, 2022 [4]	Korea	_	_	_
Pagnini et al., 2012 [22]	Italy	40	_	34.87
Pizzimenti et al., 2013 [23]	Italy	36	22/14	35.10
Stephens et al., 2016 [25]	United States	287	184/103	31.29
Wallace et al., 2014 [26]	United Kingdom	24	17/7	5 people were at stage 1 16 people were at stage 2 12 people were at stage 3 9 people were at stage 4

† ALSFRS-R Functional Assessment Scale for Amyotrophic Lateral Sclerosis—Revised. The revised version is a scale with a total of 12 items that investigate specific functions, which are frequently impaired in ALS: speech, salivation, swallowing, hand movements, cutting food and using utensils, dressing and hygiene, turning over in bed and adjusting blankets, walking, climbing stairs, and 3 items for respiratory function. For each item, a score is proposed from 0 (severe disability/impairment) to 4 (no disability), also understood as no change from before the onset of the condition. The score can vary from a minimum of 0 to a maximum of 48 [27].

**Table 4:** Tools.

	Åkerblom et al., 2020 [20]	Åkerblom et al., 2021 [14]	Chiò et al., 2012 [11]	Edge et al., 2020 [24]	Hanisch et al., 2015 [13]	Hurwitz et al., 2021 [19]	Ishida et al., 2018 [21]	Kong et al., 2021 [2]	Kwak, 2022 [4]	Pagnini et al., 2012 [22]	Pizzimenti et al., 2013 [23]	Stephens et al., 2016 [25]	Wallace et al., 2014 [26]
Face-to-face interview	X		X	X				X					
Brief Pain Inventory (BPI) [28]		X	X		X			X				X	X
ALS Functional Rating Scale-Review (ALSFRS-R) [29]		X			X			X			X	X	
Focus group				X									
Scala di valutazione del dolore NRS				X									
World Health Organization Quality-of-Life Scale (WHOQOL-BREF) [31]				X									
Anxiety and depression scale (HADS) (HADS) [30]				X								X	
Quality of Life Index (QL-Index) [32]											X		
Zung Self-Rating Depression Scale (SDS) [33]											X		
Visual analogical scale (VAS) [34]											X		
Schedule for the Evaluation of the Individual Quality of Life-Direct Weighting (SEIQoL-DW) [35]		X											
Wong–Baker Faces Pain Rating Scale (WBS) [36]							X						
Barthel Index [37]							X						
Italian Pain Questionnaire (QUID) [38]										X			
McGill Quality of Life Questionnaire (MQoL) [39]										X			
Pain Detect Questionnaire [40]													X
Survey online												X	
Chronic Pain Self-Efficacy Scale (CPSS) [41]												X	

## Data Availability

Not applicable.

## Public Involvement Statement

There was no public involvement in any aspect of this research.

## Guidelines and Standards Statement

This manuscript was drafted against the JBI Manual for Evidence Synthesis [14] and in accordance with the PRISMA ScR checklist [15].
